# Hydrogen Trapping at Fe/Cu Interfaces

**DOI:** 10.3390/ma17225671

**Published:** 2024-11-20

**Authors:** Philipp Hammer, Matthias Militzer, Vsevolod I. Razumovskiy

**Affiliations:** 1 Materials Center Leoben Forschung GmbH, Roseggerstrasse 12, 8700 Leoben, Austria; vsevolod.razumovskiy@mcl.at; 2 Centre for Metallurgical Process Engineering, The University of British Columbia, Vancouver, BC V6T 1Z4, Canada; matthias.militzer@ubc.ca

**Keywords:** hydrogen trapping, interface segregation, DFT calculations, Cu precipitate

## Abstract

Copper (Cu) in steel production can be a residual element, causing challenges during steel processing, as well as an alloying element, improving corrosion resistance and providing hardenability by nanosized precipitates. For the transition toward a green economy, increased recycling rates in steel production and alternative energy carriers, such as hydrogen, are of vital importance. As hydrogen is known for its embrittling effect on high-strength steels, this work sought to explore possible mitigation strategies for hydrogen embrittlement (HE) with the help of Cu precipitates. Hydrogen trapping at Cu/Fe interfaces following the complex phase transformations in the Cu precipitation sequence from body-centered cubic (bcc) to the so-called 9R structure to face-centered cubic (fcc) was addressed by a series of systematic density functional theory calculations. In combination with thermodynamic calculations, the pressing question regarding which of the precipitate structures was most desirable for the tackling of HE was alluded to. We found that hydrogen trapping at the Cu/Fe interfaces increased from −0.05 to −0.18 eV following the precipitation sequence. Despite this relatively weak hydrogen trapping, which was in the range of dislocations, we showed through thermodynamic calculations that fcc Cu precipitates could still contribute to lowering the risk of triggering the hydrogen-enhanced localized plasticity (HELP) mechanism of HE.

## 1. Introduction

Copper (Cu) plays an important role as an alloying element in steel production and can be used to improve the corrosion resistance of various steel grades [1,2,3] or their mechanical properties by nanosized Cu-rich particles [1,4,5,6,7,8]. On the other hand, Cu residuals introduced during the steelmaking process through scrap recycling cannot be easily removed, thus causing challenges during the later stages of steel processing [9,10,11]. The latter effects become increasingly important in view of the green economy, which relies on material recycling and the use of renewable and greenhouse-gas-emission-free energy sources [12,13,14,15]. In terms of recycling, the electric arc furnace (EAF) steelmaking process is of particular interest, which directly uses steel scrap with the aid of electricity as a carbon-free energy source [9,14] but increases the likelihood of Cu residuals in all types of steel grades. In addition, hydrogen (H) is envisioned as one of the most prominent sources of clean renewable energy in the future and the exposure of steel structures to H steadily increases [12,13,14,15]. Unfortunately, the contact between steel and H in service leads to another challenge regarding the safety of high-strength steels known as hydrogen embrittlement (HE) [15,16,17]. A possible implication of a combined effect of H and Cu on steels used in H applications deserves special attention, as it may determine the reliability of high-strength Cu-containing steels in practice [18,19].

One of the most appealing problems in this respect is related to the complex kinetics of precipitate formation in Cu containing body-centered cubic (bcc) steels, which is known to undergo several stages [1,2,6,20,21,22]. During the first stage, Cu forms coherent nanosized particles that inherit the bcc crystal lattice structure of the parent phase (α-Fe). As these particles grow, they transform into the more complex C19 structure with a twinned lattice, also referred to as 9R [2,20,21,22]. In the final stage, Cu precipitates become completely incoherent with the host bcc lattice of iron and adopt the equilibrium face-centered cubic (fcc) structure of Cu. This complex precipitation kinetics is known to have a pronounced effect on the mechanical properties of steels, such as strength, ductility, and fracture toughness, representing a topic of intense investigations [1,2,4,5,6,7,8,21,22].

In the context of HE, coherent precipitates are often used in steel design as traps for H atoms. Such traps can immobilize H atoms, and thus, prevent them from reaching and accumulating at microstructural features critical for HE (like grain boundaries (GBs) and dislocations). H accumulation at and interaction with these features is responsible for triggering either the hydrogen-enhanced decohesion (HEDE) mechanism through H interaction with GBs or the hydrogen-enhanced localized plasticity (HELP) mechanism via H interplay with dislocations. Both of these mechanisms are regarded to be among the most prominent for high-strength steels [15,23,24]. A key factor influencing the efficacy of traps to immobilize H is their trapping energy, i.e., the interaction energy of H with the trapping site, which determines how strongly H is bound to the trap. Another important metric is the amount of H traps that can be provided by such precipitates, as they are only effective in their role as long as they are not saturated with H [25]. This concept has been known for the case of coherent transition metal carbide precipitates in low-alloyed steels [15,16,25,26,27,28,29,30,31], whereas much less is known about the possible role of Cu precipitates in this process, though there are indications in the literature that some Cu precipitates may play a similar role to carbides in terms of H trapping [32,33,34,35,36,37,38,39,40,41,42,43,44].

The main reason for the limited data on H interaction with Cu nano-precipitates is related to the technical difficulties of experimental studies and theoretical description. On the experimental side, the detection of both H atoms and nano-precipitates is often found at the limits of sensitivity or resolution for modern experimental methods of microstructure characterization, making it necessary to apply multiple methods at a time to obtain a conclusive answer [45]. This makes the direct observation of H interaction with microstructural features at the nanoscale an extremely challenging task. On the computational side, the complexity of the twinned 9R structure with a nine-layer-repeated stacking sequence for Cu precipitates (see Section 2.2.3 for details) poses particular challenges for predictive density functional theory (DFT) calculations, as the necessarily large supercells used to describe this structure are at the limits of computational feasibility.

In this study, we analyzed H interactions with Cu particles in bcc iron using DFT and thermodynamic modeling. First, we constructed atomic scale interface models following crystallographic and high-resolution TEM data for three types of Cu precipitates in iron, i.e., bcc, fcc, and the 9R twinned lattice with a nine-layer-repeated stacking sequence. These models were used to determine the H-trapping energies by DFT. As a final step of our investigation, we employed these DFT results to simulate possible redistributions of H within a typical microstructure of a high-strength martensitic steel containing Cu precipitates using a thermodynamic model of H trapping. A parameter study on the influence of Cu precipitate shape and volume fractions on H redistribution closed this study, where Cu precipitate contents that stemmed from residual levels of Cu up to Cu as a deliberate alloying element in steel were considered. The findings of this investigation were used to elaborate on how one challenge of the green economy, that is, increased levels of unwanted Cu residuals due to scrap recycling, provides a possible mitigation strategy for the HE problem that is anticipated to become larger in the wake of the green energy transition.

## 2. Computational Details

### 2.1. DFT Calculations

First-principles density functional theory (DFT) calculations were performed with the Vienna ab initio simulation package (VASP, version 5.4.4) [46,47,48] using the Perdew, Burke, and Ernzerhof [49] description of the generalized gradient approximation (GGA) for the exchange–correlation functional. The valence electron configurations for the atomic potentials based on the projector-augmented wave (PAW) [50,51] method were $3p^{6}4s^{1}3d^{10}$ for Cu, $3p^{6}4s^{2}3d^{6}$ for Fe, and $1s^{1}$ for H. For all calculations, a plane-wave energy cutoff of 400 eV and spin polarization were employed and electronic self-consistency was converged to $10^{-7}$ eV/atom for the total energy. Unless stated otherwise, ionic relaxations with constant cell shape and volume were undertaken until the residual forces reached 0.02 eV/Å per atom. Electronic smearing was considered using the Methfessel–Paxton method [52], with a width of 0.2 eV to improve the convergence. The Monkhorst–Pack [53] grid sampling for Brillouin zone integration was automatized by the pymatgen [54] software package (version 2022.3.29), with the reciprocal k-point density set to 1200 ${Å}^{-3}$.

The equations of state were calculated using the Birch–Murnaghan [55] fit to the energy versus volume curves obtained by a homogeneous distribution of seven points ±15% around the minimum energy volume from Ref. [56]. Based on this fit, the equilibrium lattice parameters for $Cu_{fcc}$, $Cu_{bcc}$, and $Fe_{bcc}$ were determined as 3.635 Å, 2.890 Å, and 2.838 Å, respectively. Our results were found to be in good agreement with the existing literature reporting lattice parameters of 3.63 Å [57,58] for $Cu_{fcc}$, 2.888 Å [59] for Cub
cc, and 2.831 Å [60] to 2.84 Å [61] for $Fe_{bcc}$.

The H solution energy in the tetrahedral interstitial site of $Fe_{bcc}$ was previously determined by the authors [26] as 0.21 eV, comparing well with the values of 0.21 eV [62] and 0.23 eV [60] determined in the literature. In the same way, H solution energies in Cu were calculated, which resulted in 0.43 eV and 0.54 eV for the octahedral and tetrahedral interstitial sites in $Cu_{bcc}$, as well as 0.45 eV and 0.65 eV for the octahedral and tetrahedral interstitial sites in $Cu_{fcc}$, respectively. Our result for the octahedral interstitial site in $Cu_{fcc}$ aligns with the experimentally determined H solution energy of 0.44 eV [63], as well as the value of 0.41 eV [58] obtained by DFT calculations.

### 2.2. Interface Models

Coherent interfaces between the Fe matrix and Cu precipitates were modeled using slabs with Born–Von Kármán [64] periodic boundary conditions and a vacuum layer in the direction perpendicular to the interface. The assumption that the lattice of the softer (bulk modulus $B_{m}$ of $Cu_{fcc}$: 139 GPa, $Cu_{bcc}$: 137 GPa) coherent nanosized Cu precipitates adopts the lattice parameter of the stiffer ($B_{m}$ = 180 GPa) Fe matrix was used for the construction of all interfaces [22,59,65,66,67] and was achieved with the aid of the atomic simulation environment (ASE) [68]. All interface slabs contained a vacuum layer of 20 Å for the cases of bcc and fcc Cu precipitates and 10 Å for the reduced 9R interface. Structure visualizations were carried out using VESTA (version 3.5.5) [69] software and the POSCAR files are provided in the Supplementary Materials.

#### 2.2.1. $Cu_{bcc}/Fe_{bcc}$ Interface

**Figure 1:**
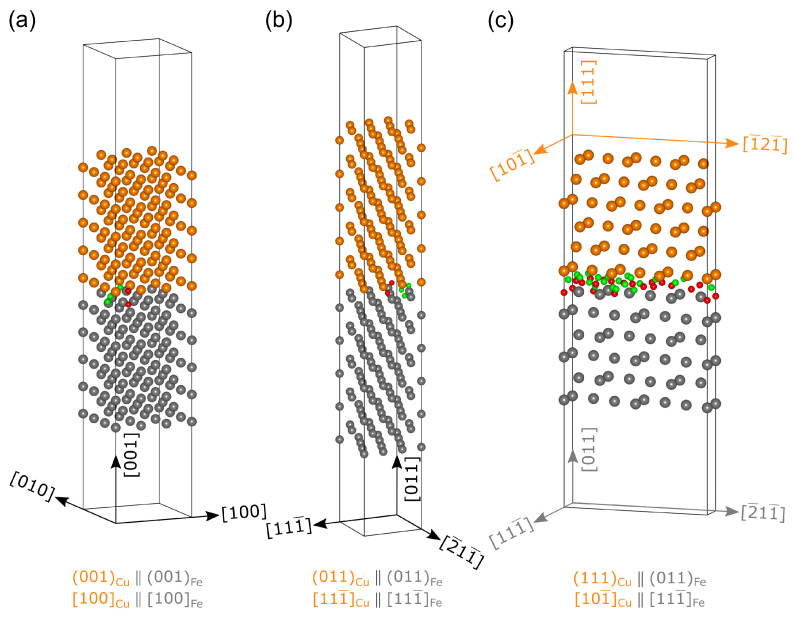
Atomic structure of (**a**) the ${\left(001\right)}_{Cu}\;\|\;{\left(001\right)}_{Fe}$ interface with $Cu_{bcc}$, (**b**) the ${\left(011\right)}_{Cu}\;\|\;{\left(011\right)}_{Fe}$ interface with $Cu_{bcc}$, and (**c**) the ${\left(111\right)}_{Cu}\;\|\;{\left(011\right)}_{Fe}$ Kurdjumov–Sachs interface with $Cu_{fcc}$. The Cu slabs (orange atoms) are stacked on top of the Fe slabs (grey atoms). The H positions investigated for later trapping energy calculations are marked with green atoms and red atoms, representing H initially placed in sites with tetrahedral and octahedral symmetries, respectively.

In the initial precipitation stage, Cu precipitates adopt the structure of the $Fe_{bcc}$ matrix phase [22,59,65,66,70,71]. The most commonly assumed orientation relationship (OR) for the corresponding interface is ${\left(001\right)}_{Cu}\;\;\|\;\;{\left(001\right)}_{Fe}$ with ${\left[100\right]}_{Cu}\;\;\|\;\;{\left[100\right]}_{Fe}$ [59]. However, the recent study of Garret and Race [66] showed that the high-energy ${\left(001\right)}_{Cu}\;\;\|\;\;{\left(001\right)}_{Fe}$ interface makes up no more than 20% of the total interface area for precipitates < 5 nm. Instead, TEM investigations [22,65,70,71,72] confirmed that the ${\left(011\right)}_{Cu}\;\;\|\;\;{\left(011\right)}_{Fe}$ interface dominates for coherent $Cu_{bcc}$ precipitates, as it displays the smallest interface energy [66,67]. In this study, we considered both aforementioned cases, i.e., the ${\left(001\right)}_{Cu}\;\;\|\;\;{\left(001\right)}_{Fe}$ with ${\left[100\right]}_{Cu}\;\;\|\;\;{\left[100\right]}_{Fe}$ OR and ${\left(011\right)}_{Cu}\;\;\|\;\;{\left(011\right)}_{Fe}$ with ${\left[11\bar{1}\right]}_{Cu}\;\;\|\;\;{\left[11\bar{1}\right]}_{Fe}$ OR, as shown in Figure 1a,b.

In the case of the ${\left(001\right)}_{Cu}\;\;\|\;\;{\left(001\right)}_{Fe}$ interface, slabs that consisted of 3×3×5 repetitions of the respective $Fe_{bcc}$ and $Cu_{bcc}$ unit cells were stacked on top of each other, with ${\left[100\right]}_{Fe}$ and ${\left[100\right]}_{Cu}$ oriented in the x-direction and ${\left[010\right]}_{Fe}$ and ${\left[010\right]}_{Cu}$ in the y-direction. The resulting interface cell displayed a lateral size of 8.51 Å in both directions and 10 layers of Fe (grey) and Cu (orange) atoms (180 total atoms), as can be seen in Figure 1a, with the Cu slab compressed by

(1) $$\varepsilon_{\left[100\right]}=\frac{a{\left[100\right]}_{Fe}^{bcc}-a{\left[100\right]}_{Cu}^{bcc}}{a{\left[100\right]}_{Cu}^{bcc}}\cdot 100=-1.80\%\;,$$

where $a{\left[100\right]}_{Fe}^{bcc}$ and $a{\left[100\right]}_{Cu}^{bcc}$ are the lattice spacings of bcc Fe and bcc Cu in their respective $\left[100\right]$ directions.

The ${\left(011\right)}_{Cu}\;\;\|\;\;{\left(011\right)}_{Fe}$ interface shown in Figure 1b was constructed by stacking 3×1×5 repetitions of the $Fe_{bcc}$ and $Cu_{bcc}$ slabs with ${\left[11\bar{1}\right]}_{Fe}$ and ${\left[11\bar{1}\right]}_{Cu}$ aligned in the x-direction and ${\left[\bar{2}1\bar{1}\right]}_{Fe}$ and ${\left[\bar{2}1\bar{1}\right]}_{Cu}$ in the y-direction, respectively. In this process, the Cu slab was compressed by −1.80% in the $\left[11\bar{1}\right]$-direction and −1.81% in the $\left[\bar{2}1\bar{1}\right]$-direction to match the respective lateral cell vectors of 7.373 Å and 6.951 Å for Fe. The direction perpendicular to the interface consisted of 10 layers of both Fe and Cu, which resulted in a total number of 180 atoms for the interface cell.

#### 2.2.2. $Cu_{fcc}/Fe_{bcc}$ Interface

Cu adopts its native fcc structure at the final stage of precipitation, forming the Kurdjumov–Sachs (K-S) OR with the $Fe_{bcc}$ matrix: ${\left(1\bar{1}1\right)}_{Cu}\;\;\|\;\;{\left(011\right)}_{Fe}$ with ${\left[110\right]}_{Cu}\;\;\|\;\;{\left[1\bar{1}1\right]}_{Fe}$ [40,73,74]. This interface was constructed using 1×3×2 repetitions of the $Cu_{fcc}$ slab with ${\left[10\bar{1}\right]}_{Cu}$ aligned in the x-direction and ${\left[\bar{1}2\bar{1}\right]}_{Cu}$ alongside the y-direction, which were stacked on top of 1×2×3 times the $Fe_{bcc}$ slab, using ${\left[11\bar{1}\right]}_{Fe}$ as the x-axis and ${\left[\bar{2}1\bar{1}\right]}_{Fe}$ as the y-axis, as shown in Figure 1c. Therefore, the Cu side of the slab was compressed by −4.40% in the ${\left[10\bar{1}\right]}_{Cu}$-direction and elongated by 4.09% in the ${\left[\bar{1}2\bar{1}\right]}_{Cu}$-direction. The resulting slab had a total of 72 atoms distributed over 6 Cu and 6 Fe layers and a cell size of $2.458\times 13.903\times 42.534\;{Å}^{3}$.

#### 2.2.3. $Cu_{9R}/Fe_{bcc}$ Interface

**Figure 2:**
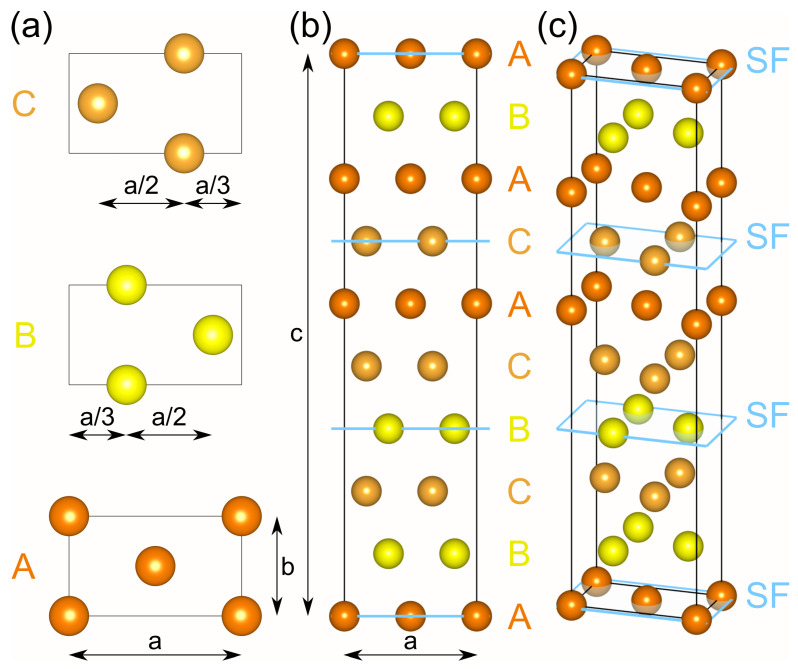
Schematic representation of the 9R structure, showing (**a**) the atom positions in relation to the cell parameters a, b, and c on the three close-packed planes (A, B, C) from the top view and the stacking sequence in the (**b**) front and (**c**) isometric views [70,75,76,78]. The stacking faults are marked by blue planes in (**b**,**c**) [70].

In the precipitation sequence $\alpha_{supeRsatuRated}\to Cu_{bcc}\to Cu_{9R}\to Cu_{fcc}$ [4,5,22,74], the initial $Cu_{bcc}$ precipitates undergo a martensitic transformation into the 9R structure once they reach a critical size of approximately 4 to 9 nm in diameter [4,21,22,75,76,77]. The bulk unit cell of the 9R structure can be idealized as orthorhombic, with lattice constants a, b, and c, although more strictly, it is reported as monoclinic, showing an angle $\beta_{0}$ between the base plane and the c-axis slightly different from 90° [22,75,76,77,78]. The three close-packed planes A, B, and C shown in Figure 2a are the building blocks of the 9R unit cell, with the stacking sequence ABC/BCA/CAB shown in Figure 2b,c [70,75,77,78]. The 9R structure can thus be considered an intermediary between the ABC/ABC stacking for fcc and the AB/AB stacking for hexagonal close-packed (hcp) structures, resulting in stacking faults after three close-packed planes, as marked with an underline in the stacking sequence above and with blue planes in Figure 2b,c [22,70,75,78]. A complete relaxation of this structure in DFT yielded a = 4.40 Å, b = 2.54 Å, and c = 18.70 Å, consistent with earlier experimental observations [20,76] of a = 4.33 Å, b = 2.50 Å, and c = 18.36 Å.

The 9R precipitate forms an interface with the $Fe_{bcc}$ following the ${\left(11\bar{4}\right)}_{9R}\;\;\|\;\;{\left(011\right)}_{Fe}$ and ${\left[\bar{1}10\right]}_{9R}\;\;\|\;\;{\left[1\bar{1}1\right]}_{Fe}$ ORs [20,21,22,65,71,75,76,78,79]. Construction of this interface at the atomic level is not as trivial as in the case of the $Cu_{bcc}/Fe_{bcc}$ and $Cu_{fcc}/Fe_{bcc}$ interfaces described earlier. Thus, the following procedure [77] was employed to construct the $Cu_{9}R/Fe_{bcc}$ interface with ${\left(11\bar{4}\right)}_{9R}\;\|\;{\left(011\right)}_{Fe}$ and ${\left[\bar{1}10\right]}_{9R}\;\|\;{\left[1\bar{1}1\right]}_{Fe}$ ORs. First, two vectors that define the ${\left(11\bar{4}\right)}_{9R}$ plane were identified as ${\left[-1\;0\;-1/4\right]}_{9R}^{T}$ and ${\left[-1\;1\;0\right]}_{9R}^{T}$, as the plane intercepted the cell axis at 1·a, 1·b, and −1/4·c. The vector perpendicular to these two vectors, and thus, normal to the ${\left(11\bar{4}\right)}_{9R}$ plane, was determined by their cross-product following Equation (2). We named this vector $z_{cart}$, as it defines the z-direction of the $Cu_{9R}$ slab and it needs to be calculated in the Cartesian rather than 9R-space for the cross-product to be valid. Consequently, each fractional vector in 9R-space has to be multiplied by the matrix of cell parameters $C={\left[\begin{array}{ccc} a & 0 & 0\\ 0 & b & 0\\ 0 & 0 & c \end{array}\right]}_{9R}$.

(2) zcart=C−1−0−149R×C−1−1−09R

To convert $z_{cart}$ back into 9R-coordinates, Equation (3) is solved for $z_{9R}$.

(3) $$C\;z_{9R}=z_{cart}$$

Finally, $z_{9R}$ can be normalized into a more convenient form using Equation (4):

(4) $$z_{9R}^{norm}=\frac{z_{9R}}{min\left\{z_{9R}^{x},\;z_{9R}^{y},\;z_{9R}^{z}\right\}}\;$$

where the superscripts *x*, *y*, and *z* denote the individual vector components in the respective axis directions. Similarly, assuming the direction given in the orientation relationship (${\left[\bar{1}10\right]}_{9R}$) will be the x-axis of the cell, the y-axis can be calculated from the cross-product of the calculated z-axis and the x-axis, again in Cartesian coordinates (Equations (5)–(7)).

(5) ycart=Cz9Rnorm×C−1−1−09R

(6) $$C\;y_{9R}=y_{cart}$$

(7) $$y_{9R}^{norm}=\frac{y_{9R}}{min\left\{y_{9R}^{x},\;y_{9R}^{y},\;y_{9R}^{z}\right\}}$$

**Figure 3:**
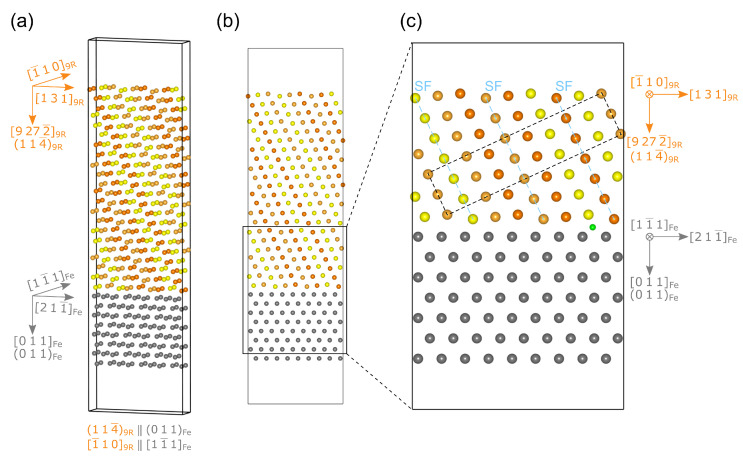
Atomic structure of the ${\left(11\bar{4}\right)}_{9R}\;\|\;{\left(011\right)}_{Fe}$ interface using the fully repeatable $Cu_{9R}$ slab in (**a**) isometric and (**b**) front views. The figure in (**c**) details the highlighted section from (**b**), showing an interface cell using only seven layers of Cu and Fe and outlining the size of a $Cu_{9R}$ unit cell, although the stacking sequence started at a C layer for convenience. Stacking fault planes [70] and the investigated H position are marked in (**c**) with blue dashed lines and a green atom, respectively. The size of the $Cu_{9R}$ unit cell is indicated by a dashed box in (**c**).

Using the aforementioned calculated lattice parameters for the $Cu_{9R}$ unit cell of a = 4.40 Å, b = 2.54 Å, and c = 18.70 Å, the $Cu_{9R}$ slab was defined by the base vectors of ${x_{9R}}^{T}={\left[\bar{1}\;1\;0\right]}_{9R}$, ${y_{9R}}^{T}={\left[1\;2.99\;1.00\right]}_{9R}\approx {\left[1\;3\;1\right]}_{9R}$, and ${z_{9R}}^{T}={\left[4.520\;13.52\;\bar{1}\right]}_{9R}\approx {\left[9\;27\;\bar{2}\right]}_{9R}$. This slab contained 396 Cu atoms, was fully repeatable in all dimensions, and aligned the ${\left(11\bar{4}\right)}_{9R}$ plane and the ${\left[\bar{1}\;1\;0\right]}_{9R}$-direction along the z- and x-axes, respectively. The iron slab with ${\left[1\;\bar{1}\;1\right]}_{Fe}$ was aligned in the x-direction, ${\left[2\;1\;\bar{1}\right]}_{Fe}$ in the y-direction, and ${\left[0\;1\;1\right]}_{Fe}$ in the z-direction could be straightforwardly generated from the bulk of $Fe_{bcc}$, and 2×3×4 repetitions of it were combined with the $Cu_{9R}$ slab to generate the interface, as shown in Figure 3a,b. Thereby, $Cu_{9R}$ was compressed by −3.24% in the ${\left[\bar{1}\;1\;0\right]}_{9R}$-direction and elongated by 1.11% in the ${\left[1\;3\;1\right]}_{9R}$-direction to produce an interface cell with a size of $4.915\times 20.854\times 78.121\;{Å}^{3}$, 8 layers (144 atoms) of Fe, and 22 layers (396 atoms) of Cu. Due to its size and number of atoms, direct DFT calculations for this interface were computationally very demanding. For the hydrogen-trapping calculations, it was thus truncated in the z-direction down to seven layers on each side of the interface, i.e., 126 atoms each for Fe and Cu, with a total of 10 Å of vacuum, as shown in Figure 3c.

### 2.3. Hydrogen-Trapping Energy

The H-trapping energy $E_{t}^{p}$ for a position *p* at a Cu interface with Fe is defined as follows [25,26,27,60]:

(8) $$E_{t}^{p}=E_{H}^{p}\left[n_{Fe};n_{Cu};1H\right]-E_{0}^{p}\left[n_{Fe};n_{Cu}\right]-\left(E_{H}^{Fe_{bulk}}\left[128Fe;1H\right]-E_{0}^{Fe_{bulk}}\left[128Fe\right]\right),$$

where $E_{H}^{p}\left[n_{Fe};n_{Cu};1H\right]$ and $E_{0}^{p}\left[n_{Fe};n_{Cu}\right]$ are the total energies of supercells with and without H in the trapping position *p*, respectively, and containing a number of $n_{Fe}$ and $n_{Cu}$ atoms. The total energies of a 4×4×4 (128 atom) $Fe_{bcc}$ supercell with and without H in the tetrahedral interstitial site are denoted as $E_{H}^{Fe_{bulk}}\left[128Fe;1H\right]$ and $E_{0}^{FE_{bulk}}\left[128Fe\right]$ and define the reference state for H [26]. By the definition of Equation (8), $E_{t}^{p}<0$ indicates that H prefers the position in trap *p* over the tetrahedral interstitial site in $Fe_{bcc}$.

### 2.4. Thermodynamic Model

Oriani [80] proposed a thermodynamic model to describe equilibrium H redistribution between the interstitial lattice sites and the available trap sites, depending on their amount (*N*) and the strength of H trapping ($E_{t}$). Over recent decades, this model was extended and generalized by a number of authors [25,81,82,83]. Here, we used the most recent version of it, as described in Refs. [25,83] in detail. This thermodynamic model assumes that each unit volume element of a material contains $N_{t}^{i}$ moles of trap types i=1,...,m and $N_{L}$ moles of interstitial lattice sites accessible for H atoms. Mass balance necessitates that the total H concentration $c_{H}$ in the system is equal to the sum of the lattice concentration $c_{L}$ and trap concentrations $c_{t}^{i}$. The fractional occupations (0≤y≤1) of the lattice and trap sites are defined as the H concentration in those sites divided by the total number of available sites and reads as $y_{L}=c_{L}/N_{L}$ and $y_{t}^{i}=c_{t}^{i}/N_{t}^{i}$, respectively. With these considerations and the definition that $E_{t}<0$ is required for H trapping, the original Oriani [80] equation becomes [25,81,83]

(9) $$\frac{y_{L}\left(1-y_{t}^{i}\right)}{y_{t}^{i}\left(1-y_{L}\right)}=exp\left(\frac{E_{t}^{i}}{RT}\right)=K^{i}\;,$$

where $E_{t}^{i}$ is the H-trapping energy of trap *i*, *R* is the ideal gas constant, *T* the absolute temperature, and $K^{i}$ the equilibrium constant for trap occupation.

Re-writing Equation (9) in terms of concentrations and considering mass balance leads to a set of equations such that

(10) $$\begin{array}{cc} \frac{c_{L}}{N_{L}}\left(1-\frac{c_{t}^{i}}{N_{t}^{i}}\right)-\frac{c_{t}^{i}}{N_{t}^{i}}\left(1-\frac{c_{L}}{N_{L}}\right)\cdot K^{i} & =0\;;i=1,...,m\\ c_{H}-c_{L}-\sum_{i=1}^{m}c_{t}^{i} & =0\;, \end{array}$$

which can be solved iteratively for the lattice ($c_{L}$) and trap concentrations ($c_{t}^{i}$), given the total H concentration $c_{H}$, the trapping energy ($E_{t}^{i}$), and the number ($N_{t}^{i}$) of traps of type *i*. The SciPy [84] software package (version 1.8.0) for Python was used to solve Equation (10).

The trap densities (number of available trap sites $N_{t}^{i}$) of the most common traps, i.e., dislocations, grain boundaries, and precipitates, can be deduced from experimental microstructure characterization data, as outlined by Turk et al. [85]. According to their work, the trap density due to dislocations $N_{disl}$ scales with the dislocation density ρ according to

(11) $$N_{disl}=N_{L}\pi {\left(5b\right)}^{2}\rho \;,$$

where $N_{L}$ is the lattice trap density of $2.04\times 10^{5}$ mol/$m^{3}$ [81,85] and *b* is the magnitude of the Burgers vector ($2.5\times 10^{-10}$ m [85]). The grain boundary trap density can be estimated by the average grain diameter $d_{g}$ following

(12) $$N_{GB}=\frac{3}{d_{g}b^{2}N_{A}}\;,$$

with $N_{A}$ representing Avogadro’s number. Likewise, the trap density associated with precipitate interfaces can be calculated using [86]

(13) $$N_{prec}=\frac{Af_{v}}{Vb^{2}N_{A}}\;,$$

where *A*, *V*, and $f_{V}$ are the precipitate interface area, volume, and volume fraction, respectively. Thus, Equation (13) can be reduced to [86]

(14) $$N_{prec}=\frac{6f_{v}}{d_{prec}b^{2}N_{A}}\;$$

for spherical precipitates with diameter $d_{prec}$ ($A=d_{prec}^{2}\pi$, $V=d_{prec}^{3}\frac{\pi }{6}$).

## 3. Results

### 3.1. Hydrogen Trapping at Cu/Fe Interfaces from DFT

The H-trapping energies at the different Cu/Fe interfaces were obtained by placing a H atom at selected interstitial interface positions *p* with tetrahedral (green atoms) or octahedral (red atoms) symmetry and having at least one Cu and one Fe atom as the nearest neighbors (see Figure 1 and Figure 3).

**Figure 4:**
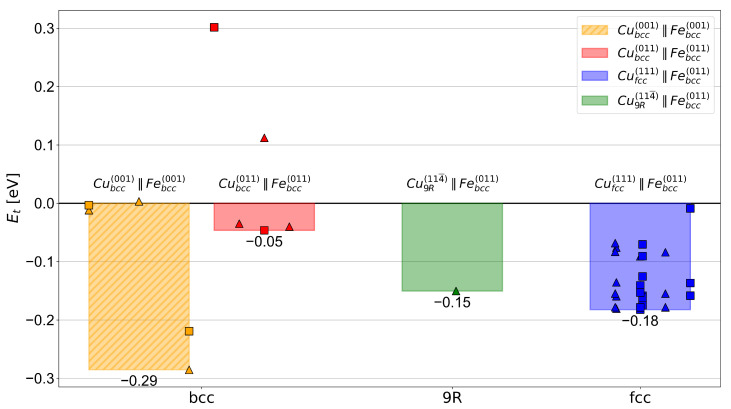
H-trapping energies at various $Fe_{bcc}$/Cu interfaces ordered according to the Cu precipitate crystal structure and precipitation sequence. The bars display the lowest trapping energy for each interface, and the individual values for each investigated H position are represented by triangles and squares for H initially placed in sites with tetrahedral and octahedral symmetries, respectively.

The trapping energies for all the investigated H positions are summarized in Figure 4. Bars in the figure mark the lowest trapping energy, i.e., the deepest trap position, found for a specific interface. For each interface, squares and triangles display the values calculated for H initially placed in positions with octahedral or tetrahedral symmetry, respectively. The alignment of the data points with respect to the bars was connected to the H position on the interface, where the left edge of each bar represents the position of the first Cu layer, while the right edge marks the first Fe layer.

For the $Cu_{bcc}^{\left(0 0 1\right)}\;\|\;Fe_{bcc}^{\left(0 0 1\right)}$ and $Cu_{bcc}^{\left(0 1 1\right)}\;\|\;Fe_{bcc}^{\left(0 1 1\right)}$ interfaces, the interstitial positions for H were straightforward to determine due to the continued bcc stacking in the supercells, as shown in Figure 1a and Figure 1b, respectively. Figure 4 illustrates that the high-energy $Cu_{bcc}^{\left(0 0 1\right)}\;\|\;Fe_{bcc}^{\left(0 0 1\right)}$ interface exhibited two positions capable of moderate H trapping. With a trapping energy of −0.29 eV, the most favored H position at this interface was one with H initially placed in a tetrahedral site at the level of the first Fe layer. In contrast, the low-energy $Cu_{bcc}^{\left(0 1 1\right)}\;\|\;Fe_{bcc}^{\left(0 1 1\right)}$ interface displayed almost no H-trapping capability, where three positions of various symmetries showed almost the same trapping energy of −0.05 eV.

All tetrahedral or octahedral interstitial positions considered for H trapping at the $Cu_{fcc}^{\left(0 1 1\right)}\;\|\;Fe_{bcc}^{\left(0 1 1\right)}$ K-S interface are shown in Figure 1c. There was a total of 13 octahedral and 18 tetrahedral non-equivalent positions that were calculated using DFT. The results show an energy range from close to zero down to −0.18 eV. As in the case of the $Cu_{bcc}\;\|\;Fe_{bcc}$ interface, the lowest trapping energy was shared by multiple H positions with both initial octahedral or tetrahedral symmetries.

The investigated $Cu_{9R}^{\left(1\;1\;\bar{4}\right)}\;\|\;Fe_{bcc}^{\left(0 1 1\right)}$ interface simulation cell that consisted of 252 atoms is displayed in Figure 3c. Here, we placed a H atom at the interstitial interface position with the largest Voronoi volume within the pre-relaxed interface structure. The considered H position is shown in Figure 3c, where it lies between the B layer of the $Cu_{9R}$ slab and a stacking fault plane (A layer). The calculated H-trapping energy for this interface was −0.15 eV, which is comparable to that of the most potent trapping sites (−0.18 eV) at the interface of fcc Cu precipitates.

### 3.2. Thermodynamic Modeling of Hydrogen Trapping at Cu/Fe Interfaces

Required input parameters for modeling the equilibrium H concentration at crystal lattice defects are the total H concentration $c_{H}$, absolute temperature *T*, H-trapping energies $E_{t}^{i}$ at all types of considered crystal lattice defects (*i*), and the corresponding trap densities $N_{t}^{i}$. In the model calculations, we assumed a total H concentration of 1 wt.ppm and room temperature of 293.15 K (20 °C). The trap densities were estimated based on a high-strength martensitic steel with 1.3 wt.-% Cu from Ref. [7] that had the following defects (traps): dislocations, prior austenite grain boundaries (PAGBs), and Cu precipitates.

Hot-rolled samples of this steel were solution-treated at 900 °C for 50 min, water-quenched, and then aged at 525 °C for 25 h, which resulted in an ultimate tensile strength of 950 MPa, and thus, a potential susceptibility to HE in the service condition, i.e., at room temperature [7]. After the heat treatment, a prior austenite grain size of 14.8 μm, a dislocation density of $4.8\times 10^{14}$ $m^{-2}$, and 1.6 vol.-% of Cu precipitates with an equivalent circle radius of ≈5 nm ($d_{Cu}\approx$ 10 nm) were determined [7]. As reported in Ref. [79], fcc Cu precipitates retain a spherical shape immediately after de-twinning from the 9R structure and grow into a rod-like shape with prolonged tempering. For the sake of comparing the influence of different precipitate shapes, we first approximated the Cu precipitates with a spherical shape of the reported equivalent radius, although the actual precipitate shape was described as rod-like with a radius of ≈3.2 nm and length of ≈20 nm [7], which is elaborated on in Section 4. No mention of the precipitate structure was made in Ref. [7], but considering the precipitate shape and comparing the reported precipitate size of ≈20 nm on the long axis with the critical size for the $Cu_{9R}\to Cu_{fcc}$ transformation (≈12–13 nm [22], 12–20 nm [6]), the Cu precipitates can reasonably be assumed to be fcc type. We therefore chose the minimum H-trapping energy of −0.18 eV—as calculated for the $Cu_{fcc}\;\|\;Fe_{bcc}$ interface—as the model parameter for the Cu precipitates. Please note that the estimated H concentration values for Cu precipitates represent the maximum concentration, as they were based on the minimum trapping energy value.

The trapping energies at dislocations and PAGBs were taken from Sato and Takai [87], who rigorously determined the de-trapping activation energies ($E_{a}$) by cryogenic thermal desorption spectroscopy. They are related to the trapping energies ($E_{t}$) required as input via the Kirchheim criterion $E_{a}≅E_{mig}-E_{t}$ [25,27,88,89], with the migration barrier $E_{mig}$ of 0.09 eV (8.68 kJ/mol) [25,26,27,90].

The trap densities for dislocations, PAGBs, and Cu precipitates could be estimated by Equations (11)–(14) using the microstructural parameters obtained by Ref. [7]. The absolute values of all input parameters used for the thermodynamic modeling of H trapping in a high-strength martensitic steel with Cu precipitates [7] are summarized in Table 1.

**Figure 5:**
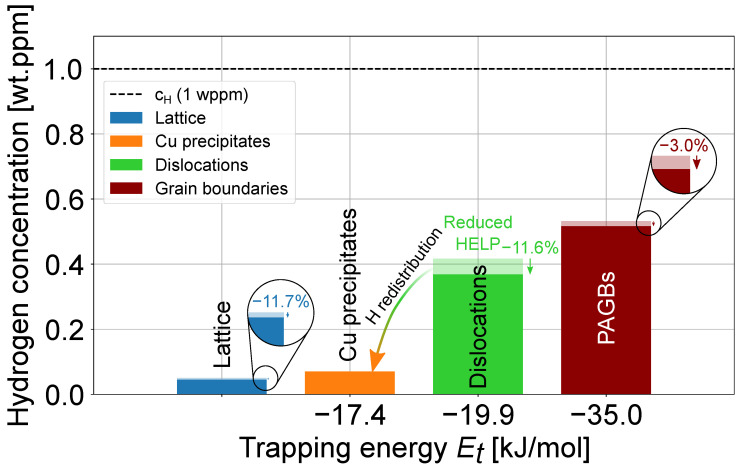
Redistribution of 1 wt.ppm H among common traps in the high-strength martensitic steel with 1.3 wt.-% Cu [7]. Transparency of the bars represents the reduction in H content due to the formation of Cu precipitates (i.e., the original H content without Cu precipitates prior to redistribution). The input parameters were taken from Table 1.

Model calculations for the equilibrium distribution of 1 wt.ppm H were conducted with and without Cu precipitates to investigate their potential impacts on the H redistribution, as represented by opaque and transparent bars in Figure 5, respectively. In the original state, H primarily occupied positions at PAGBs (0.53 wt.ppm) and dislocations (0.42 wt.ppm). With the introduction of spherical ($d_{Cu}$ = 10 nm) fcc Cu precipitates, the equilibrium H concentration at all other sites was lowered to 0.07 wt.ppm H accumulated at the $Cu_{fcc}\;\|\;Fe_{bcc}$ interface (see Figure 5a). The majority of this H was redistributed from dislocations, which reduced the H amount bound at dislocations by 11.6%. In addition, 3.0% and 11.7% of H from PAGBs and the lattice were drawn to the fcc Cu precipitates.

## 4. Discussion

The results of our DFT calculations show that the $Cu_{bcc}^{\left(0 0 1\right)}\;\|\;Fe_{bcc}^{\left(0 0 1\right)}$ interface provided the strongest traps ($E_{t}$ = −0.29 eV) of all the considered interfaces, including bcc, 9R, and fcc types. However, Garret and Race [66] showed that this relatively high energy interface accounted for less than 20% of the $Cu_{bcc}$ precipitate interface area or even may not occur at all. The authors suggest that it is rather the $Cu_{bcc}^{\left(0 1 1\right)}\;\|\;Fe_{bcc}^{\left(0 1 1\right)}$ interface that dominates for $Cu_{bcc}$ [66] precipitates in iron. Our results show, however, that this interface was much less relevant for H trapping due to its negligibly small trapping energy of −0.05 eV.

Experimental observations [22,65,70,71,72] suggest that the Cu precipitates of all types predominantly form their interfaces with the (0 1 1) plane of $Fe_{bcc}$. The H-trapping energies of −0.05 eV, −0.15 eV, and −0.18 eV calculated at these interfaces follow the precipitation sequence $Cu_{bcc}\to Cu_{9R}\to Cu_{fcc}$ [4,5,22,74], respectively (see also Figure 4). These trapping energies fall in a similar range to those of dislocations in $Fe_{bcc}$, and thus, are significantly weaker than the H-trapping energies at GBs and vacancies (see Table 2) [60]. This result implies that Cu precipitates of all types do not represent strong traps for H atoms, i.e., they may release H at close-to-ambient conditions [25]. The similarity of the corresponding trapping energies to dislocations would also suggest that it may be difficult to experimentally distinguish H trapping at these precipitates from other defects.

Despite their relatively low trapping energy, Cu precipitates may still have an important role in the distribution of H within a steel microstructure. As is evident from Equation (10), not only the trapping energy but also the trap density affect the H distribution [25,80,81,83].

The model results presented in Figure 5 show that H is expected to be mostly concentrated at dislocations and PAGBs in the martensitic high-strength steel from Ref. [7], where it is known to be the most critical for the HE sensitivity, triggering the HELP and HEDE mechanisms of HE, respectively [15,16,17,24,25]. A total of 1.6 vol.-% $Cu_{fcc}$ precipitates with a 10 nm diameter could substantially reduce the concentration of H at dislocations by introducing relatively large amounts of new traps. This microstructural change corresponded to a H concentration drop of 11.6% (from 0.42 wt.ppm to 0.37 wt.ppm) at dislocations and 11.7% at interstitial lattice sites, which reduced the potential effect of the HELP mechanism of HE in this alloy.

**Figure 6:**
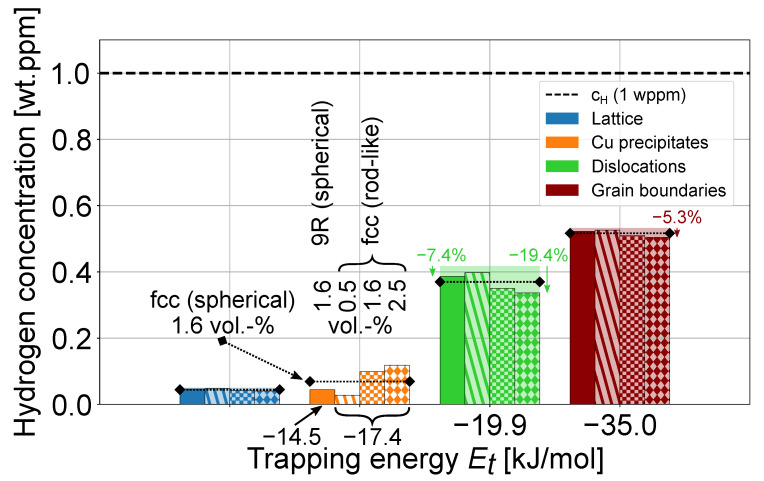
Redistribution of 1 wt.ppm H among common traps in a high-strength martensitic steel with different types of Cu precipitates [7]. For each trap, the bars from left to right represent 1.6 vol.-% spherical 9R Cu precipitates with a diameter of 5 nm (filled bar) and rod-shaped fcc Cu precipitates with a diameter of 6.4 nm; a height of 20 nm; and volume fractions of 0.5 vol.-% (striped pattern), 1.6 vol.-% (checkerboard pattern), and 2.5 vol.-% (caro pattern). Transparent bars represent the original H content without Cu precipitates and the dotted lines mark the results for spherical fcc Cu precipitates with a diameter of 10 nm (see Table 1) for comparison.

The above example considers spherical $Cu_{fcc}$ precipitates as the major Cu precipitate type that traps H in the model. However, $Cu_{9R}$ precipitates provide a much larger interface area than $Cu_{fcc}$ precipitates owing to their smaller size of $d_{9R}\approx$ 5 nm [22]. Additionally, their trapping energy (−0.15 eV = −14.5 kJ/mol) is only little smaller than that of $Cu_{fcc}$, and hence, a larger effect on hydrogen trapping would be expected. Although the provided trap density by $Cu_{9R}$ precipitates ($N_{9R}$ = $5.1\times 10^{2}$ mol/m³) is indeed almost double that for $Cu_{fcc}$ precipitates (see Table 1), their slightly smaller trapping energy interestingly still caused overall less efficient H trapping. This is illustrated by the leftmost bars in Figure 6, calculated for $Cu_{9R}$ precipitates that were assumed to have a spherical shape of diameter $d_{9R}$ = 5 nm and the same volume fraction as for the $Cu_{fcc}$ precipitates (1.6 vol.-%). The dotted line above the bars represents the results of spherical $Cu_{fcc}$ from Figure 5, and the shaded area is the original H concentration without Cu precipitates.

Conversely, $Cu_{fcc}$ precipitates are reported to have elliptical [22] or rod-like shapes [7] rather than a spherical one. The use of the effective sphere radius to estimate the precipitate trapping served as a first approximation, as a sphere displays the lowest possible surface-to-volume ratio. When approximating the rod-like $Cu_{fcc}$ precipitate as a cylinder with a diameter of 6.4 nm and a height of 20 nm [7]—which corresponded to the 10 nm equivalent spherical diameter [7] in Table 1—the trap density calculated according to Equation (13) increased to $N_{Cu,rod}$ = $3.08\times 10^{2}$ mol/m³. This led to an increased H concentration trapped at $Cu_{fcc}$ precipitates, as depicted by the bars with a checkerboard pattern for all traps in Figure 6. In addition, the volume fraction of the Cu precipitates, and thus, their contribution to H trapping, could vary due to a decreased or increased Cu content in the steel. We showcase the effect of the precipitate volume fraction on H trapping in Figure 6 by considering volume fractions of 0.5 and 2.5 vol.-% of rod-shaped $Cu_{fcc}$ precipitates with the aforementioned dimensions, as represented by the second and fourth bars with striped and caro patterns, respectively. Thus, an increased volume fraction of 2.5 vol.-% of rod-shaped fcc Cu precipitates could reduce the respective amount of H trapped at dislocations and prior austenite grain boundaries by 19.4% and 5.3% from the original concentration without Cu precipitation. In contrast, 0.5 vol.-% or less of Cu precipitates, which could be introduced by Cu residuals in the steel, only showed a minor effect on the overall H distribution.

The overall theoretical results and conclusions are supported by the earlier experimental studies of Komazaki, Koyama, and Misawa [36] and McCarroll et al. [37]. Based on atom probe tomography (APT) results on deuterium-charged samples, McCarroll et al. [37] established a trapping ranking in their steel of TiC-decorated dislocations > high-energy grain boundaries > low-energy grain boundaries > Cu precipitates, emphasizing the rather weak H trapping at Cu precipitates. A similar result was obtained by Lin et al. [38], who determined a ranking of H traps in Cu-containing steel as TiC > TiC platelets > grain boundaries > ε-copper precipitates > dislocations, similar to our results. Komazaki, Koyama, and Misawa [36] found that the fcc-structured ϵ-Cu precipitates showed a better performance than the Cu clusters/twinned 9R precipitates in a small punch test with H contents from 0.75–1.66 wt.ppm, supporting our conclusions.

Other similar studies often contained a mixed ferritic/austenitic matrix [33,34,35] or substantial amounts of other precipitate-forming elements, like Ti [36,37,38], Nb [39,40,41], V [41,42], Cr [37,39,40,43], or Mo [38,41,43], providing additional H traps, and thus, making it difficult to single out the effects of Cu precipitates and their morphology on H trapping or HE in these alloys, and therefore, they were not used for comparison with the model results presented here.

## 5. Conclusions

In this study, thermodynamic simulations to determine the H distribution and concentration at individual crystal lattice defects were conducted in order to determine the possible impact of Cu precipitates on the HE susceptibility of a selected steel microstructure. The input parameters were obtained from DFT calculations of the H-trapping energies at model Cu/Fe interfaces with different morphologies, and estimates of the corresponding trap densities were obtained from experimental input of Ref. [7]. The main conclusions are as follows:

- Despite relatively low absolute values of H-trapping energies, $Cu_{fcc}$ precipitates with a large interface area could markedly reduce the amount of H at dislocations, which lowers the risk of triggering the HELP mechanism of HE [15,24].
- The H-trapping energy obtained by the DFT calculations follows the experimentally observed evolution of the Cu precipitate structure $\alpha_{supersaturated}\to Cu_{bcc}\to Cu_{9R}\to Cu_{fcc}$ [4,5,22,74], with trapping energies of −0.05 eV, −0.15 eV, and −0.18 eV for the model interfaces of $Cu_{bcc}$, $Cu_{9R}$, and $Cu_{fcc}$ precipitates with Fe, respectively. These results are found to agree well with the experimental findings of Refs. [36,37,38,41].
- The strongest trapping energy of −0.18 eV obtained for the $Cu_{fcc}$/Fe interface is found to be in the range of dislocations, and thus, is considered rather moderate compared with GBs, vacancies, and interfaces with coherent carbides, which all have significantly lower trapping energies (i.e., are stronger traps) for H atoms in $Fe_{bcc}$ [25,60].
- Although the $Cu_{9R}$ precipitates provide a substantially increased trap density compared with the $Cu_{fcc}$ precipitates due to their smaller size, the $Cu_{fcc}$ precipitates could still trap a higher concentration of H owing to them offering slightly deeper traps. This emphasizes the importance of trapping energy differences even as small as 3 kJ/mol on the trapped concentration of H for weak-to-moderate H traps.
- While $Cu_{fcc}$ precipitates with a large interface area introduced through intentional Cu alloying might significantly contribute to the H redistribution in a steel microstructure, Cu residuals from the steelmaking process are expected to only have a minor effect on overall H trapping.

## Figures and Tables

**Table 1:** Input parameters for the thermodynamic model.

Parameter	Value	Reference	Comment
$c_{H}$	1 wt.ppm	-	Model assumption
*T*	293.15 K	-	Room temperature (20 °C)
$E_{t}^{disl.}$	−19.9 kJ/mol	[87]	8.68 kJ/mol migration barrier [25,26,27,90] $-E_{a}$
$E_{t}^{GB}$	−35.0 kJ/mol	[87]	8.68 kJ/mol migration barrier [25,26,27,90] $-E_{a}$
$E_{t}^{Cu_{fcc}}$	−17.4 kJ/mol	Table 2	−0.18 eV converted to kJ/mol
ρ	$4.8\times 10^{14}$ $m^{-2}$	[7]	Martensite tempered at 525 °C for 25 h
$d_{g}$	14.8 μm	[7]	Martensite tempered at 525 °C for 25 h
$d_{Cu}$	10 nm	[7]	Martensite tempered at 525 °C for 25 h
$f_{Cu}$	1.6 vol.-%	[7]	Martensite tempered at 525 °C for 25 h
$N_{l}$	$2.04\times 10^{5}$ mol/$m^{3}$	[81,85]	Lattice trap density for ferrite
$N_{disl}$	$4.81\times 10^{2}$ mol/$m^{3}$	Equation (11)	Dislocation trap density
$N_{GB}$	5.39 mol/$m^{3}$	Equation (12)	Grain boundary trap density
$N_{Cu}$	$2.55\times 10^{2}$ mol/$m^{3}$	Equation (14)	Trap density of $Cu_{fcc}$ precipitates

**Table 2:** Comparison of the H-trapping energies at Cu/$Fe_{bcc}^{(0 1 1)}$ interfaces with trapping data on other defects in Fe [60].

	$Cu_{bcc}$/Fe	$Cu_{9R}$/Fe	$Cu_{fcc}$/Fe	Dislocation	GB	Vacancy
$E_{t}$ [**eV**]	−0.05	−0.15	−0.18	−0.2–−0.3 [60]	−0.18–−0.61 [60]	−0.5–−0.7 [60]

## Data Availability

POSCAR files of the DFT calculations can be found in the Supplementary Materials. The raw data required to reproduce the computational results findings can be requested from the corresponding author.

## Supplementary Materials

The following supporting information can be downloaded from https://www.mdpi.com/article/10.3390/ma17225671/s1—POSCAR S1: Cu-9R_Fe_Interface_H_POSCAR.vasp, POSCAR S2: Cu-bcc-001_Fe_Interface_H_POSCAR.vasp, POSCAR S3: Cu-bcc-011_Fe_Interface_H_POSCAR.vasp, POSCAR S4: Cu-fcc_Fe_Interface_H_POSCAR.vasp.
